# Epitope Mapping for Monoclonal Antibody Reveals the Activation Mechanism for αVβ3 Integrin

**DOI:** 10.1371/journal.pone.0066096

**Published:** 2013-06-20

**Authors:** Tetsuji Kamata, Makoto Handa, Sonomi Takakuwa, Yukiko Sato, Yohko Kawai, Yasuo Ikeda, Sadakazu Aiso

**Affiliations:** 1 Department of Anatomy, Keio University School of Medicine, Shinjuku-ku, Tokyo, Japan; 2 Center for Transfusion Medicine and Cell Therapy, Keio University School of Medicine, Shinjuku-ku, Tokyo, Japan; 3 Preventive Health Examination Center, International University of Health and Welfare, Minato-ku, Tokyo, Japan; 4 The Faculty of Science and Engineering, Graduate School of Advanced Science and Engineering, Waseda University, Shinjuku-ku, Tokyo, Japan; University of Bergen, Norway

## Abstract

Epitopes for a panel of anti-αVβ3 monoclonal antibodies (mAbs) were investigated to explore the activation mechanism of αVβ3 integrin. Experiments utilizing αV/αIIb domain-swapping chimeras revealed that among the nine mAbs tested, five recognized the ligand-binding β-propeller domain and four recognized the thigh domain, which is the upper leg of the αV chain. Interestingly, the four mAbs included function-blocking as well as non-functional mAbs, although they bound at a distance from the ligand-binding site. The epitopes for these four mAbs were further determined using human-to-mouse αV chimeras. Among the four, P3G8 recognized an amino acid residue, Ser-528, located on the side of the thigh domain, while AMF-7, M9, and P2W7 all recognized a common epitope, Ser-462, that was located close to the α-genu, where integrin makes a sharp bend in the crystal structure. Fibrinogen binding studies for cells expressing wild-type αVβ3 confirmed that AMF-7, M9, and P2W7 were inhibitory, while P3G8 was non-functional. However, these mAbs were all unable to block binding when αVβ3 was constrained in its extended conformation. These results suggest that AMF-7, M9, and P2W7 block ligand binding allosterically by stabilizing the angle of the bend in the bent conformation. Thus, a switchblade-like movement of the integrin leg is indispensable for the affinity regulation of αVβ3 integrin.

## Introduction

Integrins are a family of α/β heterodimeric transmembrane cell surface receptors that mediate the cell-extracellular matrix and cell-cell interactions. The hallmark of integrin-dependent adhesive interactions is their regulation by intracellular signaling events (inside-out signaling). In addition to mediating adhesive interactions, liganded integrins initiate signals inside the cell to modify cell behavior (outside-in signaling) [1]. This integrin-mediated bidirectional signaling is closely associated with the structural rearrangement of the integrin itself. The crystal structure of the extracellular domains of αVβ3 and αIIbβ3 integrin revealed that the α chain consists of the N-terminal β-propeller domain followed by the thigh, calf-1, and calf-2 domains and that the β chain consists of the PSI, βA, hybrid, four EGF, and βT domains [2], [3]. The β-propeller and the βA domains non-covalently associate with each other to form a globular head that is observable using conventional electron microscopy (EM) [4]. By contrast, the thigh, calf-1, and calf-2 domains of the α chain and the PSI, hybrid, EGF, and βT domains of the β chain form a leg-like region, respectively. The most striking feature revealed in the crystal structure is the orientation of the head. The two legs in the crystal structure fold back at a 135-degree angle between the thigh and the calf-1 domains and between the EGF-1 and EGF-2 domains, unlike the straight leg observed using conventional EM. Consequently, the head region points downward, facing the plasma membrane. The discrepancies between these two structures were reconciled by a high-resolution EM image of the extracellular domains of recombinant αVβ3 integrin [5]. These observations revealed that αVβ3 could adopt multiple distinct structures, including the bent and the extended conformers observed in the crystal structure and conventional EM studies, respectively. Since Mn^2+^ and ligand peptide significantly increased their number, the extended form appeared to represent the high-affinity state, and the bent conformer was thought to represent the low-affinity state. Thus, the transition from one conformer to the other (the so-called switchblade-like movement) might account for the affinity regulation of the integrin. Consistent with these findings, genetically engineered αIIbβ3 constrained in the bent state interfered with the binding of macromolecular ligands, while αIIbβ3 constrained in the extended state exhibited maximal activation [6], [7]. Finally, αIIbβ3 embedded in nanodiscs underwent extension in the presence of a talin head domain that binds to the β3 cytoplasmic domain, suggesting that the switchblade-like transition actually occurs during inside-out signaling [8]. Aside from the switchblade-like movement, substantial structural rearrangement has been observed in the head region. An EM study of α5β1 integrin complexed with a fibronectin fragment revealed that the β hybrid domain swings out upon ligand binding [9]. The crystal structures of αIIbβ3 head regions complexed with short ligand peptides or ligand mimetics have provided detailed information [3], [10]. This swing-out movement is accompanied by the rearrangement of the ligand-binding and/or cation-binding loops in the βA domain, thereby regulating ligand binding. In agreement with these findings, attempts to constrain the movement of the hybrid domain in a swing-out (open headpiece) or a swing-in (closed headpiece) position revealed that this movement is critical not only for β3 integrin activation [7], [11], but also for β1 and β2 integrins [12]–[14]. Thus, these results suggest that extension and an open headpiece conformation are independently required for high-affinity ligand binding.

However, contradicting reports have suggested that integrin extension is not an essential event for ligand binding. The crystal structure of αVβ3 complexed with a small peptide ligand revealed that the bent conformer is capable of binding a ligand [15]. Understandably, αVβ3 was unable to undergo gross structural rearrangements upon ligand binding under the constraints of the crystal lattice in this experiment. However, a single particle analysis of αVβ3 complexed with a recombinant fibronectin fragment has shown that αVβ3 can bind to a macromolecular ligand when it is in a bent state in the presence of Mn^2+^ [16]. The measurement of fluorescent energy transfer between the mAb bound to the β-propeller domain and the plasma membrane in live cells revealed that αVβ3 remains in a bent conformation when activated by Mn^2+^ or an activating mutation [17]. These lines of evidence suggest that the bent conformer is capable of binding not only small ligands, but also macromolecular ligands without undergoing substantial structural rearrangements of αVβ3 integrin.

Most of the structural and/or functional studies on integrins described above have been performed using genetically manipulated molecules. Thus, it is impossible to negate the possibility that those manipulations could have an unexpected effect on the folding/gross structure of the molecule. If this were the case, interpretation of the whole data would be jeopardized. For these reasons, alternative approach is required to investigate the mechanism of integrin activation. In this study, we examined epitopes for numerous anti-αVβ3 monoclonal antibodies, some of which have been known to interfere with the αVβ3-based functions of cells. To our surprise, some of the function-blocking mAbs bound to the thigh domain of the αV chain, which does not contain a ligand-binding site. Further investigation using cells expressing human-to-mouse αV chimeras revealed that three mAbs shared an amino acid residue located above the α-genu as a common epitope. These mAbs inhibited fibrinogen binding to αVβ3-expressing cells to varying extents. To elucidate the blocking mechanism of these mAbs, αVβ3 constrained in the extended conformation was engineered. This mutant αVβ3 was highly active, compared with the wild-type, and bound fibrinogen even in the presence of Ca^2+^, which is known to inhibit αVβ3-ligand interactions. All the genu-binding mAbs failed to inhibit fibrinogen binding to the mutant αVβ3, suggesting that these mAbs block ligand binding allosterically by restricting the angle of the bend. Our findings are consistent with the hypothesis that the ligand-binding activity of integrin can be regulated by the switchblade-like movement of the leg structure of integrin centering on the genu.

## Materials and Methods

### Antibodies and Reagents

Normal mouse IgG was purchased from Sigma-Aldrich (St. Louis, MO). The mAbs against αV (CD51), β3 (CD61), or αVβ3 complex (CD51/CD61) were obtained from the following sources. Non-functional anti-β3 mAb VNR5-2 has been previously characterized [18]. Anti-β3 mAb SZ21 and anti-αV mAbs AMF-7 [19] and 69-6-5 [20] were purchased from Beckman Coulter (Fullerton, CA). Anti-αV mAbs 17E6 [21] and P2W7 [22] were purchased from Calbiochem (La Jolla, CA) and R&D Systems (Minneapolis, MN), respectively. Anti-αV mAbs P3G8 [23], M9 [24], and anti-αVβ3 complex-specific mAbs LM609 [25] were purchased from Chemicon International (Temecula, CA). Anti-αVβ3 complex-specific mAb 23C6 [26] were purchased from BD Pharmingen (San Diego, CA). Hybridomas producing anti-β3 mAb 7E3 [27] and anti-αVβ3 complex mAb 10C4.1.3 [28] were obtained from the American Type Culture Collection (Manassas, VA). RPE-conjugated goat anti-mouse polyclonal antibody was purchased from Biosource (Camarillo, CA). The synthetic peptide Gly-Arg-Gly-Asp-Ser (GRGDS) was purchased from the Peptide Research Institute (Osaka, Japan). Fluorescein-isothiocyanate (FITC) was purchased from Sigma-Aldrich (St. Louis, MO). Human fibrinogen was purchased from Enzyme Research Laboratories (South Bend, IN).

### Construction of Mutant αV cDNA Clones

**Figure 1:**
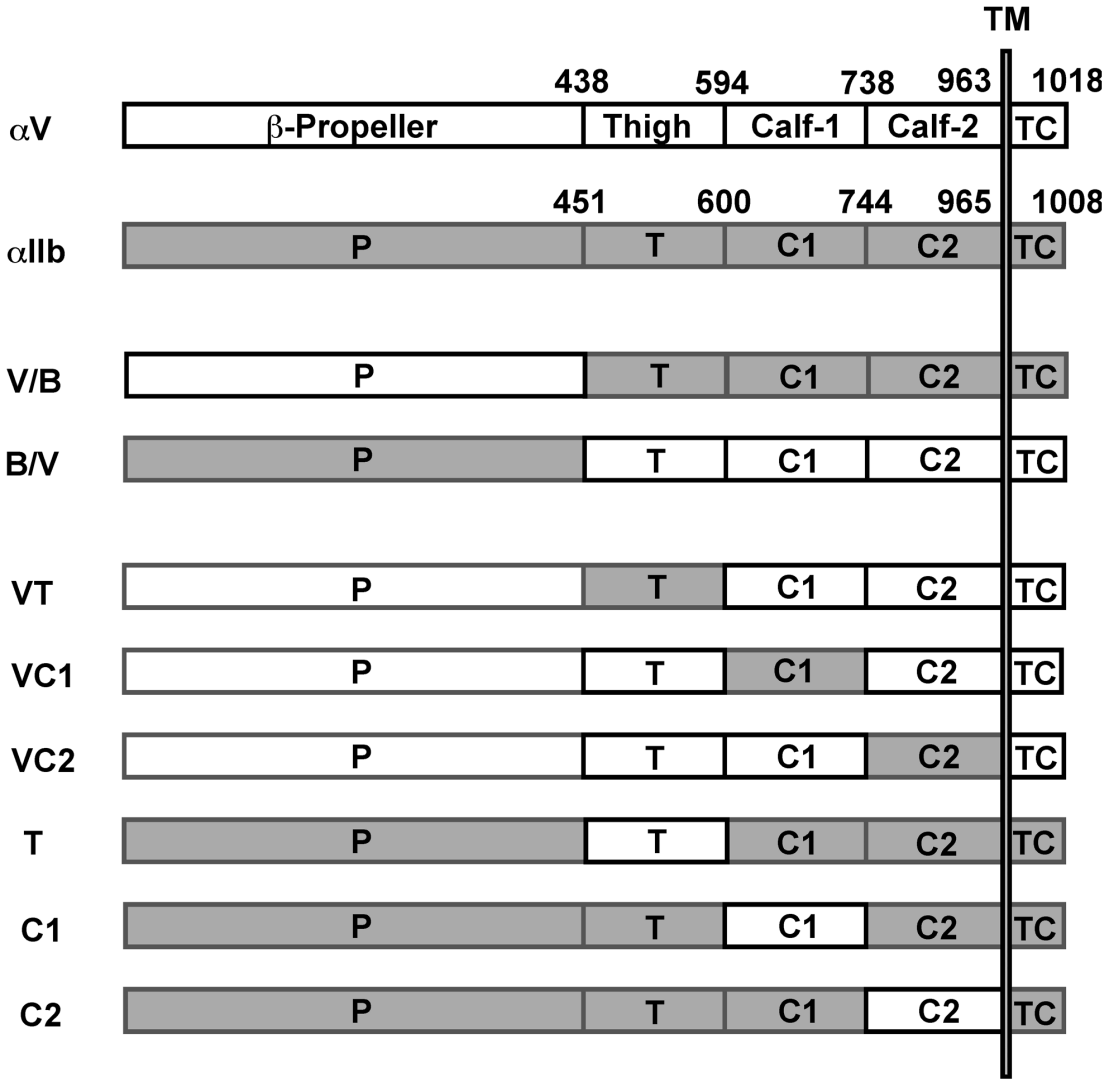
Schematic representation of αV/αIIb chimeras. The abbreviations P, T, C1, C2, and TC in the figure stand for β-propeller, thigh, calf-1, calf-2, and transmembrane-cytoplasmic domain, respectively. The numbers indicate the domain boundaries used to create the chimeras.

The full-length cDNAs for the integrin αV, αIIb and β3 subunits, which were generous gifts from Dr. Joseph C. Loftus (Mayo Clinic Scottsdale, AZ), were cloned into the mammalian expression vector pBJ-1, which was kindly provided by Dr. Mark Davis (University of California, San Francisco). The cDNAs for the αV/αIIb domain-swapping chimeras VT, VC1, and VC2 were created using the overlap extension PCR method. The cDNAs for the B/V, V/B, T, C1, and C2 chimeras have been described elsewhere [29]. The domain boundaries for each chimera were set as shown in Fig. 1. The cDNAs for the human-to-mouse αV mutants I441V, T460ICP (T460I/S462P), T460I, S462P, V486T, N492H, E496DV (E496D/L497V), Y515HN (Y515H/S516N), S520V, N524T, I527VF (I527V/S528F), I527V, S528F, L532Q, I539V, Y565Q, T571A, and I586V and the cDNA for αV-to-αIIb mutants Q456P, D457A, N458V, T460S, G465Q, A467K, L468T, and K469P and the cDNA for αV mutant Q589NAT (Q589N/H591T) were created using site-directed mutagenesis and a Transformer Site-Directed Mutagenesis Kit (BD Biosciences, San Jose, CA).

### Cell Culture and Transfection

Chinese hamster ovary (CHO)-K1 cells, obtained from the American Type Culture Collection (Manassas, VA), were cultured in Dulbecco's modified Eagle's medium (Invitrogen, Carlsbad, CA) supplemented with 10% fetal calf serum (Hyclone, Logan, UT), 1% penicillin and streptomycin (Invitrogen, Carlsbad, CA), and 1% non-essential amino acids (Sigma-Aldrich, St. Louis, MO) and maintained at 37°C in a humidified incubator supplemented with 5% CO_2_. Fifty micrograms of αV or αIIb cDNA construct was co-transfected with 50 µg of β3 cDNA construct into CHO-K1 cells using electroporation. After 48 hours, the cells were detached and used in further experiments.

### Flow Cytometry

Cells were detached with phosphate-buffered saline (PBS) containing 3.5 mM EDTA. After washing, the cells were incubated with 10 µg/mL of mAb in modified HEPES-Tyrode buffer (HTB; 5 mM HEPES, 5 mM glucose, 0.2 mg/mL bovine serum albumin, 1× Tyrode's solution) supplemented with 1 mM CaCl_2_ and 1 mM MgCl_2_ for 30 min at 4°C. After washing, the cells were incubated with an RPE-conjugated F(ab′)_2_ fragment of goat anti-mouse IgG for 30 min at 4°C. After washing, the cells were resuspended in HEPES-buffered saline (HBS; 10 mM HEPES, 150 mM NaCl, pH 7.4) containing 1 mM CaCl_2_ and 1 mM MgCl_2_; the fluorescence was then measured using a FACSCalibur (BD Biosciences, San Jose, CA).

### Fibrinogen Binding Assay

FITC labeling of human fibrinogen was performed as previously described [30]. Forty-eight hours after transfection, the cells were detached and washed once with HTB. The αVβ3-transfected cells were incubated with anti-β3 mAb VNR5-2 followed by incubation with an RPE-conjugated F(ab′)_2_ fragment of goat anti-mouse IgG. After washing, the cells were incubated with 340 µg/mL of FITC-labeled fibrinogen with or without 1 mM GRGDS peptide in HTB containing 1 mM CaCl_2_ and 1 mM MgCl_2_ or containing 2 mM MgCl_2_ and 5 µM EGTA for 2 hours at 4°C. After washing, fluorescence was measured using a FACSCalibur. The mean Fbg binding (FL1) to cell populations expressing a high β3 (FL2>1000) was calculated. Background binding in the presence of 1 mM GRGDS peptide was subtracted to obtain the specific binding. In the monoclonal antibody inhibition assays, after staining with primary and secondary antibodies, the cells were resuspended in HTB. Then an equivalent volume of 200 µg/mL mAb solution in PBS was added to yield a final concentration of 100 µg/mL. As a control, an equivalent volume of PBS was added instead of the mAb solution. Then FITC-labeled fibrinogen, MgCl_2_, and EGTA were added at concentrations of 340 µg/mL, 2 mM, and 5 µM, respectively. The specific fibrinogen binding was normalized using the expression of αVβ3 on the cell surface and by dividing the MFI (FL1) obtained in the presence of each mAb by the MFI (FL2) of the gated cell population.

### Immunoprecipitation

Biotin labeling of the cell surface protein was performed using Sulfo-NHS-Biotin (Thermo Scientific, Rockford, IL), following the manufacturer's instructions. Cells were lysed in 1 mL of lysis buffer (100 mM n-octylglucopyranoside, 20 mM N-ethyl maleimide, 1 mM PMSF, 25 mM Tris-HCl, and 150 mM NaCl, pH 7.4). After removing the insoluble material by centrifugation, the supernatant was used for further analysis. Two hundred microliters of cell lysate was precleared by adding 1 µg of mouse IgG, together with 20 µL of Protein G agarose beads. After centrifugation, the supernatant was recovered and further incubated with 1 µg of VNR5-2, together with 20 µL of Protein G agarose beads overnight at 4°C. Then the supernatant was discarded, and the remaining Protein G agarose beads were washed 3× with washing buffer (25 mM Tris-HCl, 150 mM NaCl, 0.01%TritonX-100 [pH 8.0]). After washing, the samples were subjected to 7.5% SDS-PAGE, transferred to a polyvinylidene difluoride membrane, probed with horseradish peroxidase-conjugated avidin, and detected using chemiluminescence with the West Pico Chemiluminescent Substrate (Thermo Scientific, Rockford, IL).

## Results

### Epitope for functional anti-αV mAb is localized close to the α-genu

To probe the regulatory mechanism of integrin activation, epitopes for numerous anti-αVβ3 mAbs were examined. For this purpose, we generated a series of αV/αIIb chimeras. The B/V, V/B, T, C1, and C2 chimeras have been previously described [29]. Additionally, we created the VT, VC1, and VC2 chimeras in the present study (Fig. 1). These chimeric α chains were expressed together with the wild-type β3 chain in CHO cells, and the binding of a panel of mAbs to these cells was examined using FACS. All nine anti-αVβ3 mAbs that were tested bound to cells expressing wild-type αVβ3 but not to cells expressing αIIbβ3 or to parent CHO cells, with the exception of 10C4, 23C6, and LM609, which showed a partial reactivity with cells expressing αIIbβ3 (Table 1). However, the MFI values obtained for these 3 mAbs with αIIbβ3-expressing cells were significantly lower than the MFI values obtained with αVβ3-expressing cells (data not shown). In addition, these 3 mAbs also bound to cells expressing wild-type β3 alone. These results suggested that 10C4, 23C6, and LM609 cross-reacted with the hamster αV/human β3 hybrid. The other mAbs (AMF-7, M9, P2W7, and P3G8) bound to cells expressing B/V, but not to cells expressing V/B. In contrast, 17E6 and 69-6-5 bound to cells expressing V/B, but not to cells expressing B/V (Table 1). These results clearly indicated that the epitopes for mAbs 17E6 and 69-6-5 are contained in the N-terminal β-propeller domain and that the epitopes for mAbs AMF-7, M9, P2W7, and P3G8 are contained in the C-terminal leg region, consisting of the thigh, calf-1, and calf-2 domains. On the other hand, the mAbs 10C4, 23C6, and LM609 bound to cells expressing B/V as well as those expressing V/B. The MFI obtained with B/V was equivalent to the value obtained with cells expressing β3 alone and was significantly lower than that obtained with V/B (data not shown). These results suggested that the epitopes for 10C4, 23C6, and LM609 are localized in the β-propeller domain, but not the leg region.

**Table 1:** MAb binding to cells expressing tail-swapping chimeras.

	mIgG	10C4	23C6	LM609	17E6	69-6-5	AMF-7	M9	P2W7	P3G8	SZ21
CHO	5.26	4.77	4.34	4.48	4.94	5.23	3.57	3.15	18.99	4.25	3.63
β3	2.72	55.83	70.09	61.93	3.64	4.66	2.92	3.93	4.08	5.84	49.89
αVβ3	3.47	61.09	77.73	68.92	67.32	64.4	64.17	71.05	57.59	63.15	54.52
αIIβ3	3.92	57.69	58.25	65.47	3.66	4.25	3.07	4.08	4.61	5.56	51.73
V/B	4.18	58.73	69.11	61.9	53.13	45.19	3.08	3.78	6.00	6.36	52.16
B/V	4.24	51.6	63.63	57.3	7.11	6.44	58.39	63.47	49.97	55.63	49.16

MAb binding to cells expressing wild-type human β3 (β3), wild-type human αVβ3 (αVβ3), wild-type human αIIbβ3 (αIIbβ3), tail-swapping mutants (V/B, B/V), and to parent CHO cells (CHO) was examined. The numbers represent the percentage of the cell population stained with each mAb.

Understandably, all the head-binding mAbs block ligand binding. However, some of the leg-binding mAbs also reportedly block cell adhesion, despite the fact that they bind to sites distant from the ligand-binding domain. To explore the mechanism by which these mAbs affect the αVβ3-ligand interaction, we decided to further localize the epitopes for these mAbs. To determine which domains these four leg-binding mAbs bind, individual domain sequences were exchanged between the αV and the αIIb chains. The resulting domain-swapping chimeras (T, C1, C2, VT, VC1, and VC2) were expressed together with wild-type β3 in CHO cells. The reactivity of these mAbs with αVβ3 was lost only when the αV thigh domain sequences were replaced with the corresponding αIIb sequences (VT). In contrast, these mAbs gained reactivity with αIIbβ3 when the αIIb thigh domain sequences were replaced by the corresponding αV sequences (T) (Table 2). These results clearly indicated that the epitopes for the leg-binding mAbs are entirely confined in the thigh domain of the αV chain, and not in the calf-1 or calf-2 domains.

**Table 2:** MAb binding to cells expressing domain-swapping chimeras.

	mIgG	AMF-7	M9	P2W7	P3G8	17E6	SZ21
CHO	7.83	5.86	5.72	15.51	5.78	5.91	4.8
αVβ3	5.6	68.11	78.41	63.75	67.97	76.32	59.23
VT	4.72	3.37	4.09	6.91	6.44	74.37	68.6
VC1	10.88	82.45	90.34	71.17	76.44	90.39	83.9
VC2	4.7	64.82	81.14	69.67	69.52	78.53	74.97
T	6.4	49.93	62.12	45.45	44.62	4.45	62.75
C1	5.87	3.8	4.03	5.82	6.37	4.05	61.79
C2	5.36	5.53	5.16	6.57	8.25	6.8	71.52

The numbers represent the percentage of the cell population stained with each mAb.

**Figure 2:**
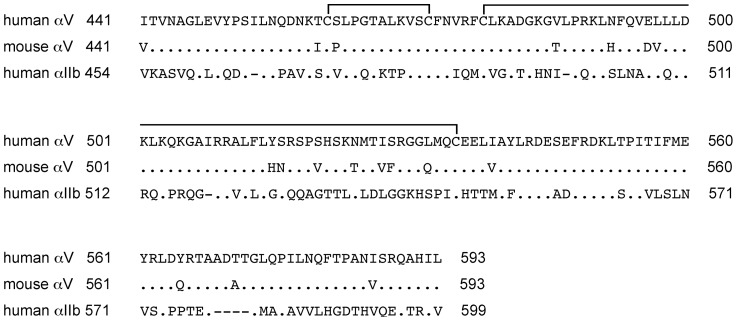
Comparison of amino acid sequences comprising the thigh domains. We show the amino acid residues 441 to 593 of the human and the murine αV chain compared with the homologous residues 454 to 599 of the human αIIb chain that differ from the human αV residues. The line connecting two Cys residues represents a disulfide link.

To further localize the epitopes for these leg-binding mAbs, short stretches of amino acid sequences in the thigh domain of human αV were replaced with the corresponding mouse αV sequences. The amino acid sequences of the mouse αV thigh domain differ from those of the human αV at 18 positions (Fig. 2). We initially created 14 human-to-mouse αV mutants and expressed them with wild-type β3 in CHO cells. As shown in Table 3, AMF-7, M9, and P2W7 failed to bind to cells expressing the T460ICP mutant, whereas P3G8 did not bind to cells expressing the I527VF mutant. None of the other mutations had a significant impact on the binding of these mAbs. The amino acid residues in the 460–462 and 527–528 regions were individually mutated to the corresponding mouse residues to identify the individual amino acid residues that were essential for the binding of these mAbs. As a result, S462P significantly blocked the binding of AMF-7, M9, and P2W7; S528F significantly blocked the binding of P3G8.

**Table 3:** MAb binding to cells expressing human-to-mouse αV mutants.

	mIgG	AMF-7	M9	P2W7	P3G8	17E6	SZ21
CHO	3.62	2.56	2.95	13.3	3.2	4.07	1.16
WT	6.8	74.11	85.85	78.21	83.47	87.07	71.41
I441V	4.15	76.76	70.66	72.64	77.61	79.97	70.78
T460ICP	5.15	5.84	3.26	7.01	70.72	77.52	65.15
T460I	5.88	62.11	63.27	54.8	56.43	70.81	64.01
S462P	5.52	14.66	3.75	17.17	66.41	76.18	66.88
V486T	11.93	72.65	78.35	71.75	64.14	82.23	71.1
N492H	10.04	70.78	79	70.14	72.01	82.49	73.56
E496DV	14.9	73.25	76.95	67.31	75.08	75.01	58.72
Y515HN	8.47	59.79	67.31	59.04	65.69	66.81	50.75
S520V	8.33	65.13	72.76	61.29	73.55	71.38	60.68
N524T	12.98	60.75	69.31	58.6	65.03	66.37	57.98
I527VF	9.55	74.93	78.94	59.25	9.26	84.31	81.13
I527V	3.12	75.57	78.3	64.88	77.78	83.6	67.47
S528F	5.32	80.94	82.19	76.5	10.59	85.83	78.1
L532Q	26.68	62.2	77.22	58.2	62.38	76.84	71.26
I539V	13.13	73.98	67.01	57.12	68.84	76.24	64.92
Y565Q	11.64	70.1	77.22	64.52	71.32	80.13	72.63
T571A	27.84	68.76	69.91	54.89	59.55	75.22	75.28
I586V	9.99	70.96	78.21	61.16	70.96	68.54	76.34

The numbers represent the percentage of the cell population stained with each mAb. Bindings significantly lower than those for 17E6 or SZ21 are marked in red.

**Figure 3:**
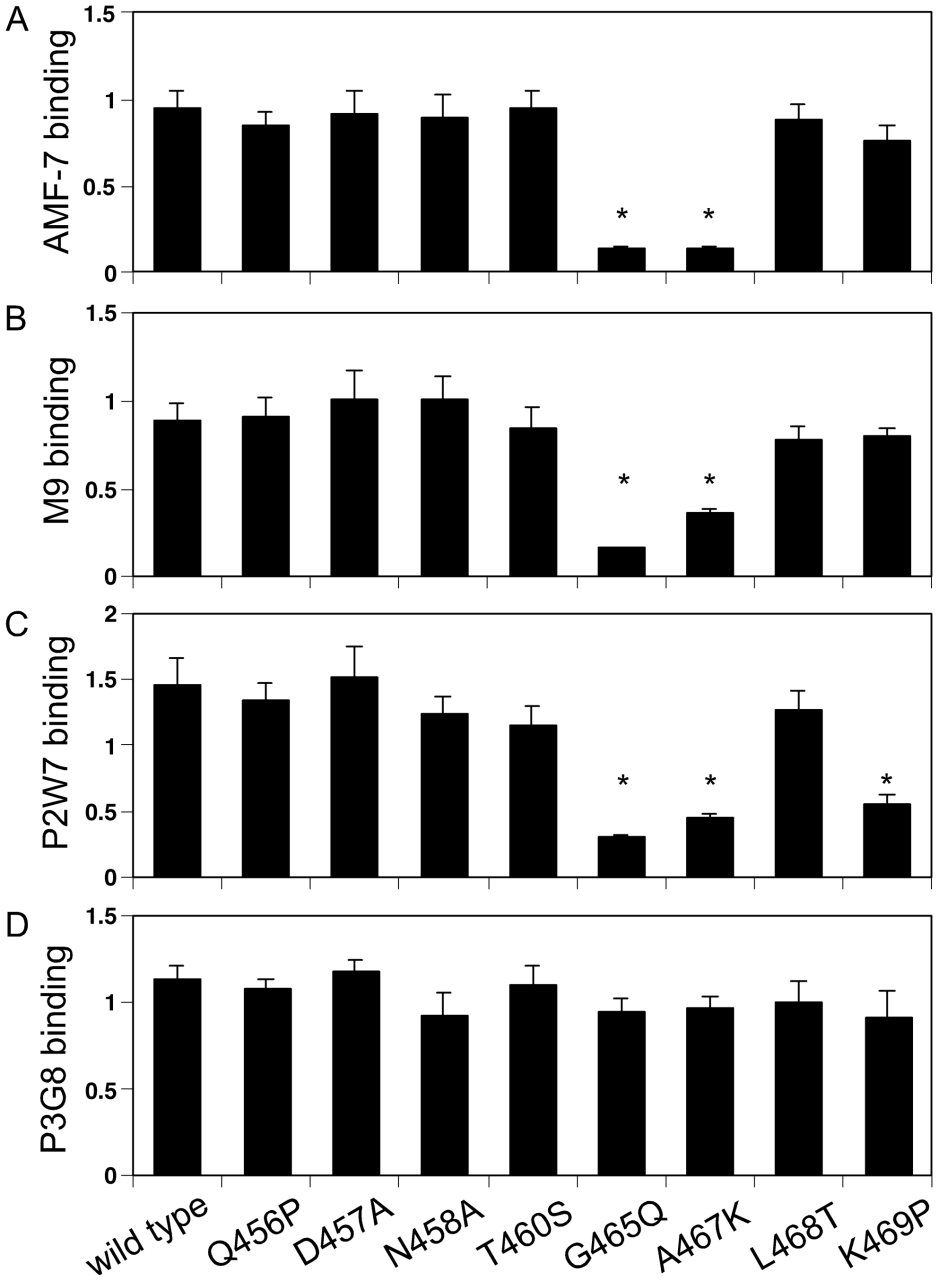
Effect of αV-to-αIIb mutation on the binding of the leg-binding mAbs. The amino acid residues 457–467 of the αV chain were individually mutated to the corresponding αIIb residues. The mutant αV was transiently expressed together with wild-type β3 in CHO cells, and the binding of mAbs to these cells was examined by FACS. The ratio of the MFI obtained from the whole cell population with each mAb to that obtained with 7E3 is shown as normalized binding. Asterisks indicate binding that was less than 50% of the wild type.

**Figure 4:**
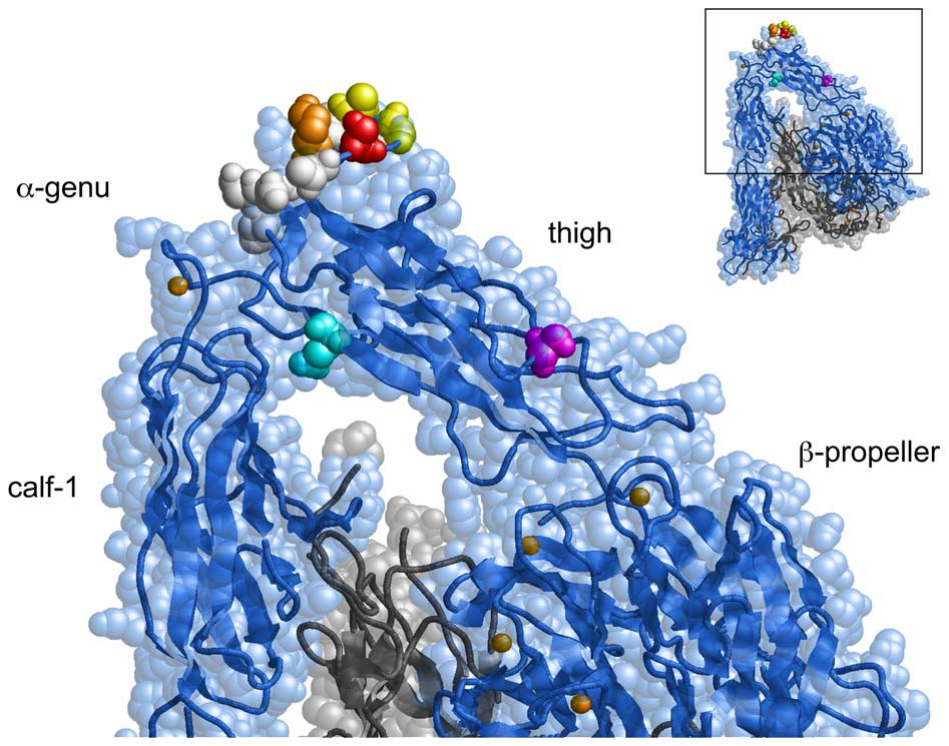
Locations of the critical residues for mAb binding in the three-dimensional αVβ3 structure. The crystal structure of the αV chain is shown by the blue spacefill representation, with its backbone shown by the ribbon. The β3 chain is shown by the gray ribbon. The epitope residues Ser-462 and Ser-528 are highlighted in red and magenta, respectively. Gly-465 and Ala-467 — which create a binding interface for AMF-7, M9, and P2W7 — are highlighted in yellow. Lys-469, important for P2W7 binding, is highlighted in orange. Residues that did not affect mAb binding when mutated are highlighted in white (see text). Gln-589 is highlighted in cyan. Note that Ser-462 is located above the α-genu, while Ser-528 is located on the side of the thigh domain distal to the α-genu.

Sharing a common epitope does not necessarily imply that AMF-7, M9, and P2W7 bind to exactly the same site. The binding interfaces of AMF-7, M9, and P2W7 were further investigated using αV/αIIb chimeras. Although Ser-462 is located in a disulfide-bonded loop, any involvement of other residues in the binding of these mAbs was impossible to determine using human-to-mouse chimeras. For this reason, amino acid residues 456–469 were mutated to the homologous residues in αIIb (Fig. 2) and expressed together with wild-type β3 in CHO cells. As shown in Fig. 3, these αV-to-αIIb mutations affected mAb binding differently. The binding of AMF-7, M9 and P2W7 were all impaired by G465Q and A467K. In addition, K469P significantly attenuated P2W7 binding. In the crystal structure, the disulfide-bonded loop including Ser-462 is located above the α-genu (Fig. 4). Although Asp-457, Ala-467, and Lys-469 were separated in the primary structure, they were all located close to Ser-462 in the tertiary structure. On the other hand, Ser-528 was located on the side of the thigh domain distal to the α-genu (Fig. 4).

### MAbs that bind to the α-genu affected ligand binding differently

**Figure 5:**
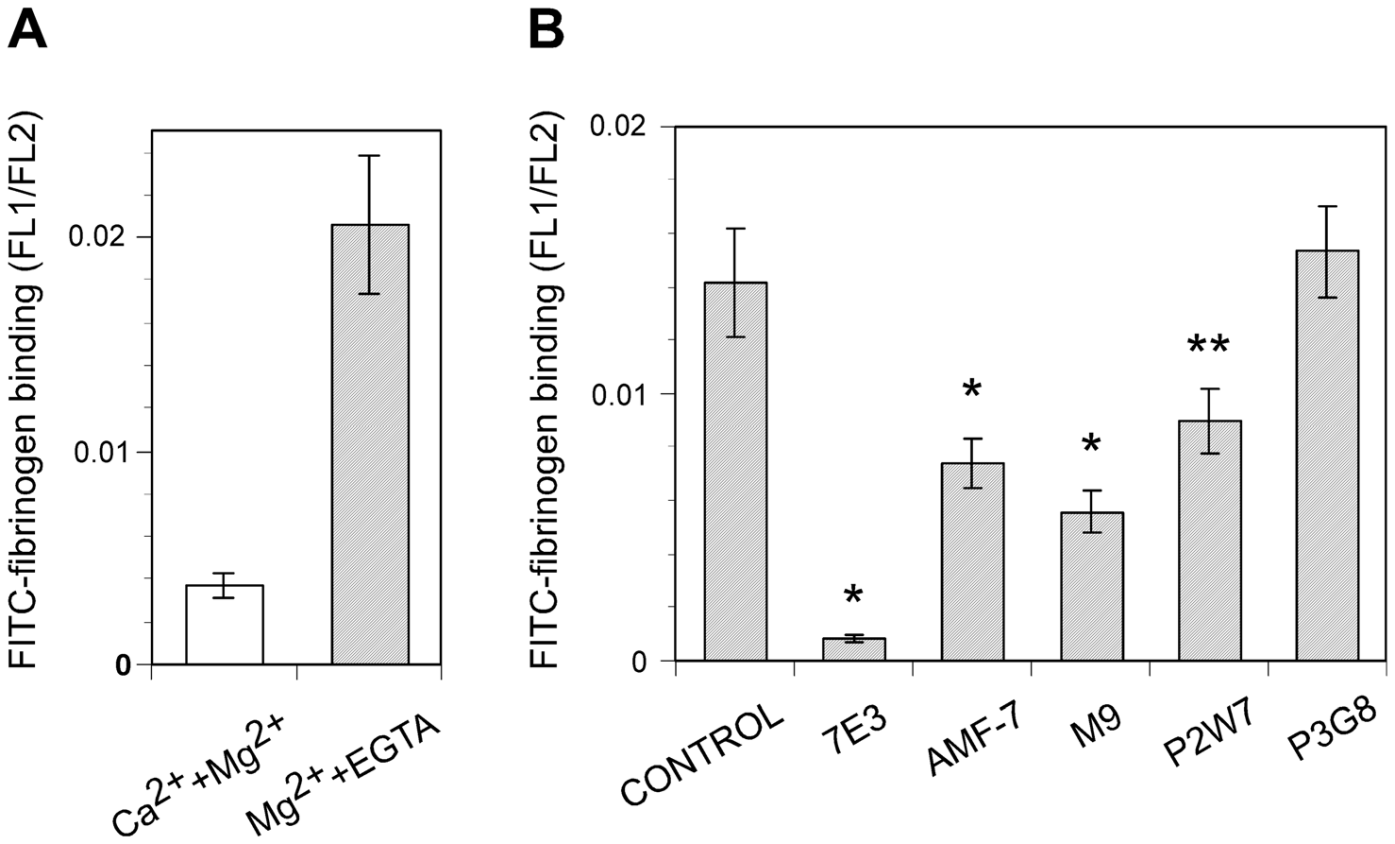
Effect of anti-αVβ3 mAbs on fibrinogen binding. A. FITC-fibrinogen binding to cells expressing αVβ3 in the presence of 1 mM Ca^2+^ and 1 mM Mg^2+^ (open column) or in the presence of 2 mM Mg^2+^ and 5 µM EGTA (hatched column) is shown. The ratio of the MFI (FL1) to the MFI (FL2) in the gated cell population was used to normalize the binding with the expression of αVβ3 on the cell surface. B. FITC-fibrinogen binding to cells expressing wild-type αVβ3 in the presence of 2 mM Mg^2+^ and 5 µM EGTA was examined. Binding in the presence of 100 µg/mL of the indicated mAb is shown in the hatched column. An equivalent volume of PBS, instead of the mAb solution, was included to obtain the control binding. The asterisks indicate statistically different binding abilities, compared with the control (*P<0.01, **P<0.05).

**Figure 6:**
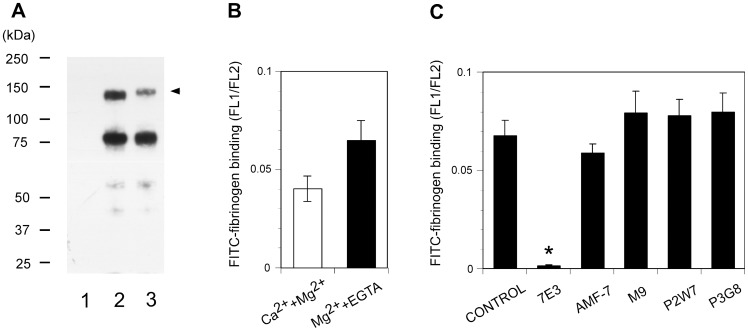
Effect of integrin extension on the function of the anti-αVβ3 mAbs. A. SDS-PAGE analysis of αVβ3 expressed on CHO cells. Cell lysates from biotin-labeled cells expressing αVβ3 were immunoprecipitated with anti-αV mAb P2W7. The precipitates were subjected to a 7.5% non-reducing gel and visualized using chemiluminescence. The positions of the molecular weight markers are shown on the left side of panel. Lane 1, parent CHO; lane 2, wild-type αVβ3; lane 3, Q589NAT. Note that the αV carrying the Q589NAT mutation (arrowhead) migrated more slowly than the wild type. B. FITC-fibrinogen binding to cells expressing αVβ3 carrying the Q589NAT mutation was examined as described in Fig. 4A. Binding in the presence of 1 mM Ca^2+^ and 1 mM Mg^2+^ (open column) or in the presence of 2 mM Mg^2+^ and 5 µM EGTA (solid column) is shown. C. The effect of the anti-αVβ3 mAbs on fibrinogen binding to cells expressing αVβ3 carrying the Q589NAT mutation was examined, as described in Fig. 5B. Among the mAbs, only 7E3 significantly inhibited binding.

**Figure 7:**
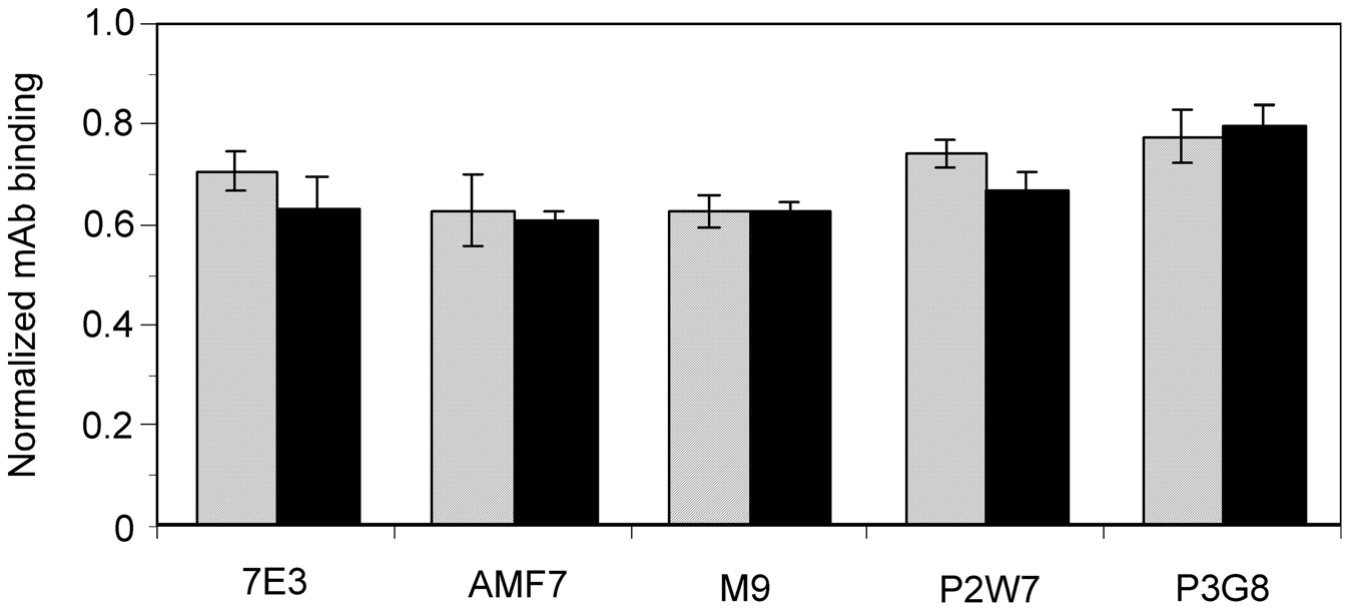
Effect of integrin extension on the binding of the anti-αVβ3 mAbs. Wild-type αV or αV carrying the Q589NAT mutation was transiently expressed together with wild-type β3 in CHO cells. The binding of function-blocking mAbs to these cells was examined by FACS. The MFI obtained from the whole cell population with each mAb was normalized by the MFI obtained with an anti-αV mAb 17E6 that represents the αV expression. There was no significant difference in the binding of leg-binding mAbs in cells expressing Q589NAT (solid column) as compared with cells expressing wild-type αV (hatched column).

To confirm the effect of these leg-binding mAbs on αVβ3-ligand interactions, fibrinogen binding to αVβ3-transfected cells was examined in their presence. FITC-labeled fibrinogen was incubated with αVβ3-expressing cells, and bound fibrinogen was measured using FACS. However, cells expressing the wild-type αVβ3 did not bind fibrinogen in the presence of 1 mM Mg^2+^ and 1 mM Ca^2+^. Ca^2+^ is known to block αVβ3-ligand interactions, while Mg^2+^ supports such interactions. For this reason, 2 mM Mg^2+^ was utilized together with 5 µM EGTA to remove residual Ca^2+^ from the buffer. Under this cation condition, wild-type αVβ3 exhibited modest fibrinogen binding (Fig. 5A). Next, the effect of the anti-αVβ3 mAbs was examined using the same cation condition. The function-blocking anti-β3 mAb 7E3, which binds to the ligand-binding βA domain, significantly inhibited fibrinogen binding to cells expressing wild-type αVβ3. In contrast, anti-αV mAb P3G8 did not affect the binding. All three mAbs that bind above the α-genu blocked binding, albeit not as potently as 7E3 (Fig. 5B). A high-resolution EM study of recombinant αVβ3 revealed that the leg of the αV undergoes a bending/extending movement at the genu. These results suggest the possibility that the three mAbs might regulate the activity of αVβ3 by stabilizing the angle of the bend in favor of the bent conformation. To examine whether this situation actually occurs, fibrinogen binding was examined on cells expressing αVβ3 constrained in the extended conformation. To constrain αVβ3 in the extended state, an N-glycosylation site consisting of an N-X-T/S motif was introduced at amino acid residue Gln-589 of αV, which is located at the back of the genu (Fig. 4). The resulting Q589NAT mutation was thus expected to prevent αVβ3 from adopting a bent conformation. An SDS-PAGE analysis of the αVβ3 that immunoprecipitated with the anti-αV mAb revealed that the αV chain with the Q589NAT mutation migrated slightly more slowly than the wild-type αV, indicating the attachment of an extra glycan to the mutant (Fig. 6A). The homologous Q595NTT mutation in αIIb has been shown to constitutively activate αIIbβ3 [7]. Likewise, cells expressing the Q589NAT mutation exhibited robust fibrinogen binding, compared with cells expressing wild-type αVβ3 (Fig. 6B). Finally, the effect of anti-αVβ3 mAbs on fibrinogen binding to Q589NAT was examined using the same cation condition. As in the wild type, anti-β3 mAb 7E3 significantly blocked binding. In contrast, the mAbs AMF-7, M9, and P2W7 all failed to inhibit binding, as did P3G8 (Fig. 6C). It appeared possible that the Q589NAT mutation might directly affect the binding of these mAbs. To exclude this possibility, the reactivity of these mAbs was compared between cells expressing wild-type αVβ3 and cells expressing Q589NAT mutation. The result confirms that the Q589NAT mutation did not have any effect on the binding of AMF-7, M9, P2W7, or P3G8, as the binding of 7E3 (Fig. 7).

## Discussion

We previously reported that extended αIIbβ3 had a high affinity for fibrinogen, whereas bent αIIbβ3 had a low affinity [7]. This previous study was based on a comparison of genetically engineered αIIbβ3, in which the three-dimensional structure was constrained either in the extended or bent conformation. However, these artificially engineered conformers do not necessarily represent native conformations that wild-type proteins adopt during physiological activation. For this reason, we used another approach to investigate the role of integrin extension in affinity regulation. In the present study, we showed that 1) the epitope for a group of anti-αV mAbs is located above the α-genu at which the leg of the integrin molecule bends, 2) these mAbs had a partial blocking effect on the αVβ3-ligand interaction, 3) constraining αVβ3 in its extended conformation resulted in robust activation, and 4) genu-binding inhibitory mAbs failed to block ligand binding to the extended αVβ3 molecule. Our results are consistent with the switchblade hypothesis (in which integrin extension increases the affinity for ligands), rather than the dead-bolt theory (in which integrin activation is restrained by the βA/βT interaction, the disruption of which activates integrin without causing substantial extension).

Among the nine anti-αVβ3 mAbs that we tested, five of them — 10C4, 23C6, LM609, 17E6, and 69-6-5 — bound to the β-propeller domain, which composes a ligand-binding site with the βA domain. Consistently, these mAbs reportedly block the function of αVβ3 integrin [20], [21], [25], [28]. On the other hand, among the four mAbs that bound to the thigh domain, AMF-7 reportedly inhibits cell adhesion [19] and M9 inhibits cell migration [24]. In contrast, P3G8 does not inhibit cell attachment to adhesive ligands [23], and no functional role has been reported for P2W7. We found these results surprising, since the thigh domain is located at a distance from the ligand-binding site. A fibrinogen binding study confirmed that AMF-7, M9, and P2W7 have a blocking effect on ligand binding, while P3G8 does not have any such effect (Fig. 5B). The effects of these mAbs were statistically significant. Notably, the function-blocking mAbs recognized the amino acid residue Ser-462, which is located within the disulfide-bonded loop above the α-genu, as a common epitope (Fig. 4). How can these genu-binding mAbs block ligand binding even though they bind at a site distant from the ligand-binding site? The genu-binding mAbs might block fibrinogen binding directly, depending on the orientation of the bound mAb. However, experiments using αV/αIIb domain-swapping chimeras suggest that the epitopes for the genu-binding mAbs are contained entirely within the thigh domain. These results indicate that the orientation of the bound mAbs relative to the bound ligand or the ligand-binding sites remains the same, regardless of the bent/extended states. If true, the genu-binding mAbs would likely block the extended αVβ3 as well. However, the results of our mAb blocking study on Q589NAT mutation suggest otherwise (Fig. 6C). These results seemed to indicate that the mAbs affected ligand binding via an allosteric mechanism, presumably by restricting the angle of the bend. However, we lack conclusive evidence that Q589NAT mutation actually adopts an extended conformation at this point. Although our conclusion is based on a reasonable assumption, further investigation is required to confirm our claim. Consistent with this idea, the binding interfaces of these mAbs were located close to the possible thigh/calf-1 interface in the extended conformation (Fig. 4). This mechanism might partly explain why the blocking effects of these mAbs were relatively weak compared with that of 7E3, which binds to the βA domain and inhibits ligand binding competitively [31]. Interestingly, epitope residues for an activating mAb against the α chain of αLβ2 integrin have been mapped to the back of the thigh domain, which is shielded in the bent conformation [32]. These previous results complement the present findings.

Numerous studies have shown that integrin extension is a prerequisite for integrin activation. A genetic approach in which integrin is constrained in a bent or extended conformation or in an open or closed headpiece conformation in αIIbβ3 and αLβ2 has indicated that both extension and an open headpiece are required for the binding of macromolecular ligands [6], [7], [13]. In agreement, another approach in which the αXβ2 conformation was constrained using functional mAbs has shown that an extended and open headpiece conformation represents a high-affinity state [14]. These studies clearly indicated that the extended conformation has a higher affinity for ligands than the bent conformation. Our results showed that αVβ3 was no exception. A substantial increase in fibrinogen binding was observed when αVβ3 was constrained in the extended conformation (Fig. 6B).

In contrast, several studies have suggested that the above is not true for αVβ3 integrin. Among them, the most compelling evidence showed that recombinant soluble αVβ3 could bind a fibronectin fragment while in a bent conformation [16]. This finding suggests that a bent and closed headpiece conformation represents a high-affinity state in αVβ3, which is completely opposite to the above-described results. The key to resolving this discrepancy may reside in the experimental conditions used in the experiments. As Blue et al. pointed out, whether integrin extension is required for ligand binding depends on the size of the ligand [6]. This situation probably arises because small ligands can gain access to a ligand-binding site in the proximity of the plasma membrane more easily than large ligands when the integrin is in its bent conformation. In other words, extension may regulate the accessibility of the ligand to integrin on the cell surface. If this is the case, extension might no longer be required in the absence of a plasma membrane. In agreement with this hypothesis, the recombinant αVβ3 lacked a transmembrane-cytoplasmic domain and the FNIII_7-10_ fragment used in their experiment was relatively small and bound to αVβ3 at the C terminus, which would not interfere with the plasma membrane in the first place. However, considering the fact that FNIII_7-10_ is located in the middle of the molecule, not at the terminus, the presence of the plasma membrane would hinder the binding of a native fibronectin molecule in the bent conformation. Thus, extension would be required for αVβ3 on the cell surface to interact with native fibronectin. Another important point is how the integrins are activated. In most experiments, Mn^2+^ is used to “activate” αVβ3 integrin. Although Mn^2+^ has been shown to induce extension in recombinant soluble αVβ3 [5], this might not be true for αVβ3 expressed on the cell surface with an intact interaction between the transmembrane-cytoplasmic domains of the α and the β subunits. Consistent with this idea, the height of αIIbβ3 reconstituted in liposomes did not change after Mn^2+^ activation [33]. In our experiments, the expression of an anti-LIBS epitope, which is preferentially expressed in liganded integrins, was not observed after Mn^2+^ activation, suggesting that extensive structural rearrangement did not occur because of Mn^2+^ alone (unpublished data). A comparison of the crystal structures of liganded and unliganded αVβ3 has revealed that the rearrangement of the cation/ligand binding sites occurs without a swing-out in the presence of Mn^2+^ [2], [15]. A similar rearrangement was accompanied by the swing-out of the hybrid domain in a liganded αIIbβ3 headpiece that lacked the lower leg region in the presence of Ca^2+^/Mg^2+^ [3]. These results suggest that although an open headpiece conformation might be required for activation in Ca^2+^/Mg^2+^, such a conformation might not be necessary in Mn^2+^. If this is the case, a bent and closed headpiece conformation could bind macromolecular ligands as long as the proximity of the plasma membrane did not prevent ligand access. Thus, integrins could bind ligands in the bent and closed headpiece conformation under specific experimental conditions. However, in physiological settings, extension and open headpiece conformation are both indispensable for high-affinity ligand interactions. It would be interesting to examine whether a closed headpiece conformer binds ligand in the presence of Mn^2+^. In addition, the direct interaction of a soluble bent conformer with a ligand would have to be established to substantiate the hypothesis described above.

Our results and those of others indicate that the extended conformation has a higher affinity for ligands than the bent conformation, and nothing more. Although integrins fixed in a completely bent or extended conformation can be engineered and examined, the intermediate conformations between these two extremes are difficult to recreate experimentally. For this reason, no information is available regarding how much extension is needed to allow ligand binding. In other words, complete extension may not be necessary for initial ligand binding. This idea may explain why no substantial differences in the fluorescent donor-accepter separation distance between the fluorescent dye-labeled membrane and the fluorescent dye-labeled mAb bound to the β-propeller domain were detected between wild-type and a constitutively active mutant of αVβ3 [17]. Instead, an external force applied to the head region may be required to accomplish extension, as simulations of the molecular dynamics of αVβ3 have suggested [34]. Such forced extension may greatly stabilize integrin-ligand interactions [35]. Taken together, the present findings suggest that the switchblade-like movement of the integrin leg regulates the αVβ3-ligand interaction.
